# Size and Sex-Dependent Shrinkage of Dutch Bees during One-and-a-Half Centuries of Land-Use Change

**DOI:** 10.1371/journal.pone.0148983

**Published:** 2016-02-10

**Authors:** Mikail O. Oliveira, Breno M. Freitas, Jeroen Scheper, David Kleijn

**Affiliations:** 1 Departamento de Zoologia, Universidade Federal do Pará, UFPA/MPEG, Belém, PA, 66075–110, Brazil; 2 Departamento de Zootecnia, Universidade Federal do Ceará, Pici Campus, Fortaleza, CE, 60356–000, Brazil; 3 Resource Ecology Group, Wageningen University, Droevendaalsesteeg 3a, 6708 PB, Wageningen, the Netherlands; 4 Animal Ecology Team, Alterra, P.O. Box 47, 6700 AA, Wageningen, The Netherlands; 5 Plant Ecology and Nature Conservation Group, Wageningen University, Droevendaalsesteeg 3a, 6708 PB, Wageningen, the Netherlands; Scientific Research Centre, Slovenian Academy of Sciences and Arts, SLOVENIA

## Abstract

Land-use change and global warming are important factors driving bee decline, but it is largely unknown whether these drivers have resulted in changes in the life-history traits of bees. Recent studies have shown a stronger population decline of large- than small-bodied bee species, suggesting there may have been selective pressure on large, but not on small species to become smaller. Here we test this hypothesis by analyzing trends in bee body size of 18 Dutch species over a 147-year period using specimens from entomological collections. Large-bodied female bees shrank significantly faster than small-bodied female bees (6.5% and 0.5% respectively between 1900 and 2010). Changes in temperature during the flight period of bees did not influence the size-dependent shrinkage of female bees. Male bees did not shrink significantly over the same time period. Our results could imply that under conditions of declining habitat quantity and quality it is advantageous for individuals to be smaller. The size and sex-dependent responses of bees point towards an evolutionary response but genetic studies are required to confirm this. The declining body size of the large bee species that currently dominate flower visitation of both wild plants and insect-pollinated crops may have negative consequences for pollination service delivery.

## Introduction

Bees are functionally important in both natural ecosystems and man-made landscapes. Bees are the main pollinators of most wild plant species and insect-pollinated crops [1,2,3]. The current decline of both wild bees and the managed Western honey bee (*Apis mellifera*) in significant parts of their range [4,5] has inspired many studies to examine both the drivers and the consequences of bee decline. These studies show that a combination of habitat destruction, agricultural intensification and the associated loss of floral resources, climate change and exposure to pesticides and pathogens drives the loss of bee populations [6,7,8] and may negatively affect the pollination services they provide [9,10,11]. Little is known, however, about the ability of bees to adapt to changes in their environment. Individuals have the ability to ameliorate, through modifications in their behavior, morphology, or physiology, the negative consequences of altered environmental conditions [12]. Furthermore, changing environmental conditions can lead to heritable, genetic changes in populations of animals over relatively short time scales [13,14].

Bee body size is closely related to flight range and mobility and therefore access to the floral resources on which bees rely for food for themselves and their offspring. Large females can also carry greater pollen or nectar loads and visit more flowers per unit time than small females [15,16,17]. At the same time, large bees require more pollen and nectar for maintenance and reproduction than small bees [18]. Along with factors such as morphology of host plant flowers [19], this trade-off between costs and benefits will influence what body size results in the highest fitness benefits for individuals of a particular species. Under conditions of environmental change, this could give rise to size-dependent selective pressure. Recent studies have found that during the past century large-bodied bee species have been declining faster in number and distribution than small-bodied species [20,21]. The contrasting responses of differently sized species implies that in large species small individuals have a selective advantage over large individuals while this is not the case in small species. A possible mechanism could be that, because less food is required to produce small offspring than large offspring, when food availability is limiting small individuals can produce more offspring than large individuals. This could then result in large-bodied bee species becoming smaller over time while body size of small species would not change significantly. So far, shrinking body size has mainly been related to global warming (e.g. [22]) but the evidence for relations between body size of bees and changing temperatures is inconclusive [23,24]. As far as we know, no studies exist demonstrating trends in body size that differ with the size of species.

Here, we test the hypothesis that large-bodied bee species have become smaller more rapidly than small-bodied species during the past century. Because bees generally display sexual size dimorphism, with females being significantly larger than males, we furthermore investigate whether this hypothetical relationship is the same in male and female bees. We additionally tested whether global warming might explain or influence trends in bee body size. We do this by examining trends in bee body size over a 147-year period of changing environmental conditions in the Netherlands. During this time, land-use in the Netherlands gradually changed from being dominated by the low-input and varied farming systems that provided ample resources to most Dutch bee species, to being dominated by intensive and monotonous farming systems that are increasingly inhospitable to bees. Throughout this period, bees have been collected by entomologists making it possible to trace back changes in bee body size. We measured, in entomological museum collections, body sizes of more than 4,500 bee specimens from 18 bee species collected across the Netherlands and analyzed whether trends in bee body size were dependent on initial size and sex of species.

## Results

**Fig 1:**
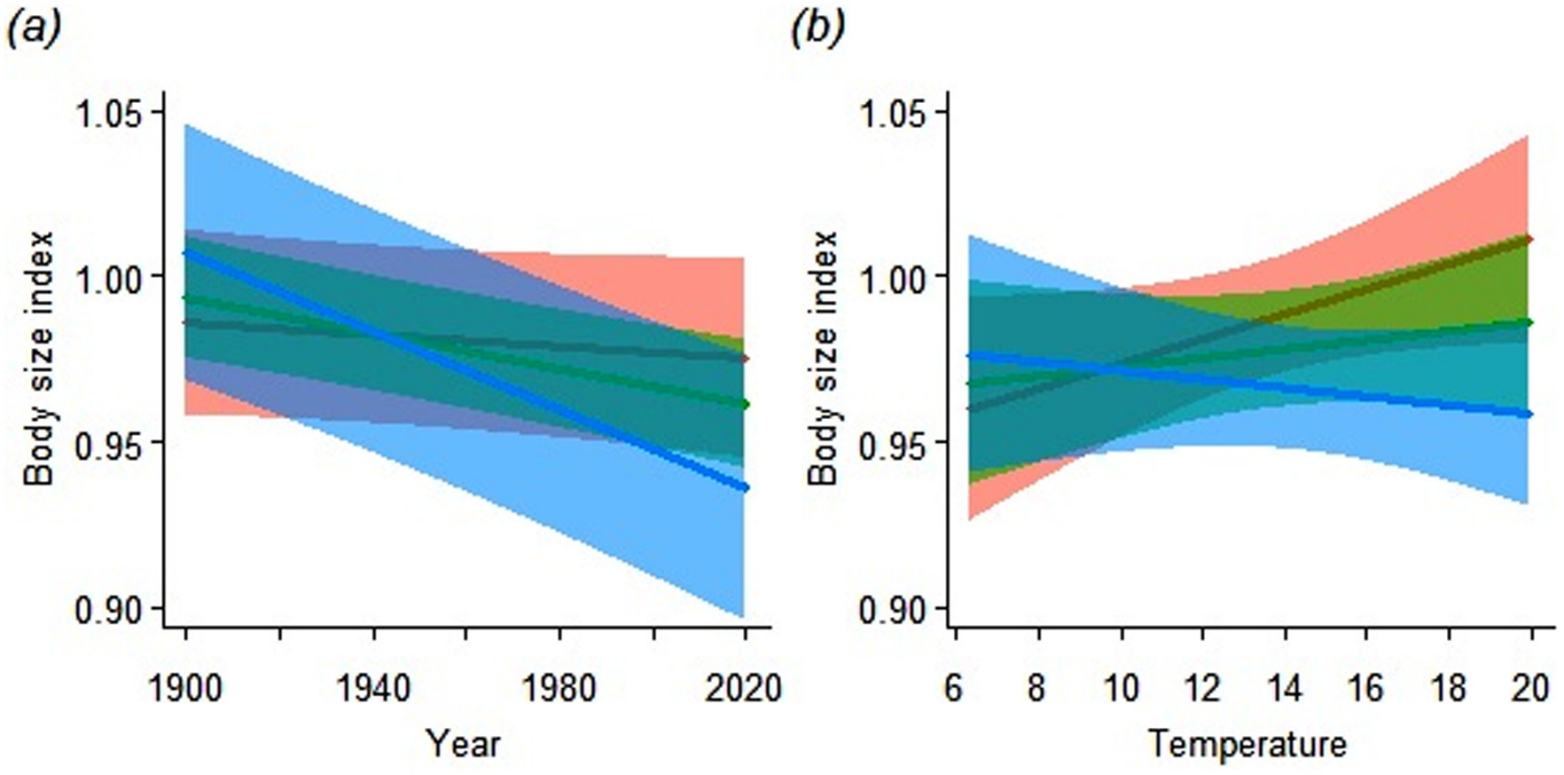
Conditional partial regression plots for the additive interacting effects of (a) year and initial body size and (b) temperature and year during flight period on female bee body size. Red, green and blue lines show cross-sections taken at the 10^th^, 50^th^ and 90^th^ percentile of the other interacting variable: (a) red = initial body size of 1.43 mm, green = 2.83 mm, blue = 5.37 mm; (b) red = the year 1919, green = 1958, blue = 2001. Shaded areas indicate 95% confidence intervals. As initial body size was defined as the mean body size in the period 1866–1899, the body size index in 1900 does not necessarily equal 1.

Overall, female bee body size declined 3.0% between 1900 (predicted body size index ± se = 0.993 ± 0.010) and 2010 (0.963 ± 0.010). However, body size of larger bees declined more strongly during this period than smaller bees (interaction between initial size and year: χ^2^_(1)_ = 7.94, *P* = 0.005, Fig 1a): the largest species became 6.5% smaller (predicted body size 1900 = 1.006 ± 0.022; 2010 = 0.941 ± 0.022) whereas the body size of the smallest species remained more or less stable, declining only 0.5% (1900 = 0.983 ± 0.017; 2010 = 0.979 ± 0.018). The non-significant three-way interaction between year, initial size and temperature (Table 1) indicates that, although temperatures during the flight periods of bees have generally increased since 1900 (linear mixed model with temperature as response variable, year as fixed factor and random intercepts and slopes for species: *t*_2051_ = 17.21, *P* < 0.001), the differential pattern for small and large bees was not affected by changes in temperature during the flight periods of the different species. Temperature did play a role in explaining changes in bee body size in general, but this effect depended on year (interaction between temperature and year: χ^2^_(1)_ = 5.94, *P* = 0.015) and was additive to the interaction effect of year and initial body size (Table 1). Body size increased with temperature early in the study period, but decreased with temperature in more recent years (Fig 1b).

**Table 1:** Results of likelihood ratio tests for interaction effects of Year, Initial body size and Temperature during flight period on bee body size. Significant effects are shown in bold.

	χ^2^	*P*
***Females***		
Year * Initital size * Temperature	0.68	0.409
Year * Initital size	7.94	**0.005**
Year * Temperature	5.94	**0.015**
Initital size * Temperature	1.13	0.288
Year	22.01	**< 0.001**
Initial Size	0.02	0.885
Temperature	0.51	0.477
***Males***		
Year * Initital size * Temperature	2.00	0.157
Year * Initital size	0.14	0.709
Year * Temperature	5.56	**0.018**
Initital size * Temperature	3.13	0.077
Year	3.58	0.058
Initial Size	0.16	0.689
Temperature	0.16	0.685

In general, male bees declined 1.3% between 1900 (0.981 ± 0.015) and 2010 (0.968 ± 0.016). In contrast to females, changes in body size of males did not depend on the initial body size of the different species, but only depended on the interaction between year and temperature (Table 1).

## Discussion

Over the course of the past one-and-a-half centuries, females of large bee species became significantly smaller but females of small species maintained stable body sizes, thus confirming our hypothesis of size-dependent species shrinkage. However, our hypothesis was rejected for male bees, since males of both large and small species showed little change in body size. Over the full range of the study period (1870–2010), the estimated body size of *Bombus terrestris* queens, both the largest species in our dataset and one of the most common bees in the Netherlands, declined with 8.2%. For comparison, the length of the Dutch human male population increased by 10.2% between 1865 and 1997 [25].

The height increase in Dutch males is considered to be one of the most spectacular growth responses ever recorded for human populations [26]. It is largely attributed to the fact that in the past one-and-a-half centuries, living conditions of people in the Netherlands improved spectacularly. In the 1860’s people, particularly in the overpopulated cities, were malnourished and heavily exposed to infectious and water-borne diseases [25]. Currently, people in the Netherlands have an abundant and varied diet and diseases no longer impact body growth. The almost similar trend in the opposite direction in large female bees suggests that habitat quality for this group has declined drastically. The significantly different patterns for large and small species and the different trends between sexes within large species, could point towards food limitation as the key factor driving the shrinkage of bees. Of all bees, females of large species have the highest nectar and pollen demands as they not only need to maintain their large bodies but they also need to provision the brood cells of their large offspring [18]. Male bees don’t assist with offspring provisioning and mainly need nectar and pollen for body maintenance, resulting in much lower resource demands, even for large species.

Metabolic rates scale with temperature [27] and global warming could be an alternative explanation for a reduction in bee body size through time, especially if bees cannot compensate with greater resource intake [22]. We don’t think global warming could explain the observed responses in bee body size for three reasons. First, mass-specific metabolic rates of small-bodied organisms tend to be higher than those of larger-bodied organisms [28] which would, if anything, have resulted in larger body size reductions in small bees than in large bees. This is the opposite of what we have observed. Second, temperature induced responses cannot explain the differential body size trends of males and females of the same bee species since metabolic rates do not differ between sexes. Third, accounting for the relationship between body size and temperature in the analyses did not change the differential body size trends of large and small female bees (Table 1). In fact, in the early part of the study period, warm years resulted in larger rather than smaller bees in the following year (Fig 1b), suggesting that in years with more warm and sunny days the improved foraging conditions more than compensate for higher metabolic rates. Only during the last part of the study, the period coinciding with the most rapid land-use change, did warmer years correlate with slightly smaller body sizes.

The persistent decline in body size of large bee species could have a genetic basis. When resource availability is frequently interrupted by disturbances, small genotypes are more likely to succeed in producing offspring and passing on their genes than large genotypes [29]. For male bees being large may represent fitness advantages because it is positively related to the possibility to mate with females [30,31]. In males, but not in females, there may therefore be opposing selection forces at work, which could explain why we only observed the size-dependent shrinkage in female bees. The quantity and quality of pollen and nectar used to provision offspring affects offspring size [32]. A continuous decline in floral resources throughout the study period could be an alternative explanation for the observed shrinkage, but resource-mediated phenotypic responses are the same for male and female bees (T. Bukovinszky et al. pers. comm.) and do not explain the observed differences between the sexes. However, our study did not examine the processes underlying the observed patterns and so far we can only speculate about the exact mechanisms. Studies examining the genetic basis of variation in body size and how this is related to food availability and temperature are required to shed more light on this.

Changing body size of large female bees could have significant consequences for ecosystem functioning and service delivery. In temperate regions, most of the dominant bees in semi-natural habitats and crops are large species of bumblebees (*Bombus* spp.*)*, digger bees (*Anthophora* spp.) and mining bees (*Andrena* spp.) [33]. Shrinkage of large bees could result in morphological mismatches between plant and pollinator species [34]. While wild plant species might have co-evolved with smaller body size of their dominant pollinators, crop breeding generally does not take pollination efficiency into account. This could have contributed to the observed decreased effectiveness of crop pollination over the past century [10].

This study demonstrates size- and sex-dependent changes in bee body size in the Netherlands over a 147-year period. Although the exact mechanisms are yet to be demonstrated, there is little evidence that they are related to global warming and the most logical explanations for these changes are related to declining and increasing unpredictability of floral resources caused by large-scale land-use change. Since land-use change and global warming are closely related in large parts of the world, studies examining body size trends in relation to global change should take land-use change-related factors into account.

## Materials and Methods

### Sampling of bees in entomological collections

In the entomological collections of the Naturalis Biodiversity Center at Leiden, Netherlands, we sampled specimens collected between 1866 and 2013 of 18 different species from seven genera: *Andrena* (*A*. *barbilabris*, *A*. *bicolor*, *A*. *nitida*), *Anthophora* (*A*. *plumipes*, *A*. *retusa*), *Bombus* (*B*. *pascuorum*, *B*. *pratorum*, *B*. *terrestris*), *Halictus* (*H*. *rubicundus*, *H*. *tumulorum*), *Lasioglossum* (*L*. *leucozonium*, *L*. *calceatum*, *L*. *villosulum*), *Megachile* (*M*. *centuncularis*, *M*. *leachella*, *M*. *maritima*) and *Osmia* (*O*. *caerulescens*, *O*. *bicornis*), see S1 Table. For the *Bombus* species, we only sampled queens and males. For genera represented by two species we sampled a small and a large species and for genera represented by three species we selected a small, an intermediate and a large species. No live bees were collected or killed in the field for this study and permission to sample the collections was granted by Naturalis.

Of each specimen we measured the inter-tegular distance (ITD, the distance between the two insertion points of the wings) as a proxy for body size [35]. To guarantee equal distribution of samples over the entire study period we defined sampling intervals: before 1900, 1900–1919, 1920–1939, 1940–1959, 1960–1979, 1980–1999 and the period after 2000. We aimed to sample 20 individuals of each sex and species from each sampling interval. Ultimately we sampled a total of 4,510 specimens, on average 131 females and 120 males per species, see S1 Table. All sampled specimens had been collected in The Netherlands, and sampling locations were distributed more or less regularly over the country.

To quantify changes in temperature during the study period, we calculated for each year the average daily mean temperature during the flight period of each bee species, for males and females separately. Temperature during flight period was the mean daily temperature during the flight period of each species in the year prior to the year of collection of the specimens. The reason for using temperature in the year before sampling is that offspring body size is positively related to the amount of pollen and nectar with which mother bees provision their eggs (e.g. [32,36,]). Furthermore, the size of the offspring is influenced by the temperature during larval development, with higher temperatures resulting in smaller adults [37]. For most Dutch bee species larval development overlaps with the flight period of the parents. The size of the bees that emerge from hibernation in a particular year should therefore be related to the temperature in the previous year, rather than the year of collection.

The flight periods of bees were determined using the national bee distribution database of European Invertebrate Survey—The Netherlands [38]. Based on the records in this database, we calculated the 10th and 90th percentile of the recording day (1 January = 1) for each bee species and defined the number of days between the 10th and 90th percentiles as the length of the flight period [21]. Average mean daily temperatures during the flight periods of the examined bees were calculated for each year using long-term (1901–2012) meteorological data obtained from weather station De Bilt [39], located in the center of the Netherlands. No meteorological data was available for the period before 1901.

### Analysis

We performed separate analyses for males and females. To facilitate comparison among species, we used an index for body size as response variable. For each species, this index expressed body size measured after 1900 relative to the initial body size of the species. The initial body size of bee species was quantified as their mean body size in the period before 1900 (1866–1899) and was used as a measure of the intrinsic interspecific difference in body size among species. We used linear mixed models to test whether body size decreased more rapidly in large-bodied bees than in small-bodied bees, which would be supported by a significant negative interaction between the fixed factors year and initial body size. Our full model furthermore included the average mean daily temperature during the flight period of bees as fixed factor, and all the two- and three-way interactions with the other fixed factors, to examine the influence of global warming on changes in body size.

We did not use phylogenetic regression since phylogenetic analyses are controversial [40] and phylogenetic trees for bees are unresolved [41]. Instead, we initially used genus, with species nested in genus, as random factor to account for potential non-independence among closely related bee species [20], and included random slopes for the effect of year. However, models not including genus as random factor produced similar results and provided better fit to the data. Our final models therefore only included species as random factor, with random slopes for the effect of year.

Our main aim was to test the hypothesis that the extent to which body size has declined differs among differently sized bee species. We therefore used traditional null hypothesis significance testing to evaluate our hypothesis. Significance of fixed effects was assessed using backward model simplification and likelihood ratio tests. We visually inspected diagnostic plots to check for normality and homoscedasticity of residuals. All analyses were performed using R 3.1.2 [42].

## Supporting Information

S1 Appendix Dataset on which the study is based. (TXT)

S1 Table Overview of species sampled. (DOCX)
