# Supplementary material for: Size and Sex-Dependent Shrinkage of Dutch Bees during One-and-a-Half Centuries of Land-Use Change
Source: PLoS One. 2016 Feb 10;11(2):e0148983. doi: 10.1371/journal.pone.0148983 (PMC4749255; doi:10.1371/journal.pone.0148983)
Supplement: S1 Table — (DOCX) [file pone.0148983.s002.docx]

**Supporting Information S1: Overview of species sampled.**

| Species | Sampled individuals | | Range in years | | Average size (mm) | |
| --- | --- | --- | --- | --- | --- | --- |
|  | Female | Male | Female | Male | Female | Male |
| *Andrena barbilabris* | 140 | 140 | 1877 - 2010 | 1884 - 2007 | 2.28 | 1.71 |
| *Andrena bicolor* | 140 | 110 | 1876 - 2007 | 1873 - 2002 | 1.65 | 1.25 |
| *Andrena nitida* | 135 | 134 | 1900 - 2009 | 1870 - 2009 | 2.96 | 2.19 |
| *Anthophora plumipes* | 115 | 94 | 1879 - 2009 | 1879 - 2003 | 4.55 | 4.34 |
| *Anthophora retusa* | 117 | 136 | 1875 - 2001 | 1874 - 2001 | 3.79 | 3.61 |
| *Bombus pascuorum* | 151 | 164 | 1878 - 2006 | 1880 - 2000 | 4.84 | 3.89 |
| *Bombus pratorum* | 136 | 165 | 1868 - 2005 | 1868 - 2005 | 4.73 | 3.51 |
| *Bombus terrestris* | 126 | 111 | 1867 - 2007 | 1879 - 2003 | 5.48 | 3.85 |
| *Halictus rubicundus* | 142 | 113 | 1872 - 2002 | 1866 - 2001 | 2.05 | 1.72 |
| *Halictus tumulorum* | 119 | 110 | 1874 - 2012 | 1874 - 2001 | 1.10 | 1.00 |
| *Lasioglossum calceatum* | 139 | 120 | 1866 - 2012 | 1866 - 2002 | 1.77 | 1.65 |
| *Lasioglossum leucozonium* | 128 | 113 | 1866 - 2008 | 1867 - 2013 | 1.92 | 1.56 |
| *Lasioglossum villosulum* | 116 | 74 | 1868 - 2002 | 1878 - 2000 | 1.38 | 1.17 |
| *Megachilidae centuncularis* | 127 | 116 | 1868 - 2004 | 1879 - 2000 | 2.96 | 2.69 |
| *Megachilidae leachella* | 122 | 95 | 1869 - 2013 | 1869 - 2006 | 2.84 | 2.49 |
| *Megachilidae maritima* | 122 | 108 | 1871 - 2002 | 1871 - 2006 | 3.35 | 3.29 |
| *Osmia caerulescens* | 138 | 118 | 1871 - 2002 | 1869 - 2002 | 1.98 | 1.67 |
| *Osmia bicornis* | 138 | 138 | 1869 - 2002 | 1866 - 2008 | 2.70 | 2.23 |
